# Measurements of Intra- and Extra-Cellular 5-Methyltetrahydrofolate Indicate that *Bifidobacterium Adolescentis* DSM 20083^T^ and *Bifidobacterium Pseudocatenulatum* DSM 20438^T^ Do Not Actively Excrete 5-Methyltetrahydrofolate *In vitro*

**DOI:** 10.3389/fmicb.2017.00445

**Published:** 2017-03-21

**Authors:** Markus Kopp, Kerstin Dürr, Matthias Steigleder, Thomas Clavel, Michael Rychlik

**Affiliations:** ^1^Chair of Analytical Food Chemistry, Technische Universität MünchenFreising, Germany; ^2^ZIEL Institute for Food and Health, Core Facility Microbiome/NGS, Technische Universiät MünchenFreising, Germany; ^3^Institute of Medical Microbiology, RWTH University HospitalAachen, Germany

**Keywords:** bifidobacteria, gut microbiota, folate synthesis, folate bioavailability, stable isotope dilution assay, LC-MS/MS

## Abstract

Certain intestinal bifidobacteria have the ability to synthesize folates. *In vitro* experiments revealed a high production, cellular accumulation, and release of reduced folate vitamers like 5-methyltetrahydrofolate and tetrahydrofolate in folate-free medium (FFM). However, it is still unclear to which extent synthesized folates are polyglutamylated and probably not available for transport, and if they are actively released by excretion. To address these questions, we characterized intra- and extra-cellular pteroylmonoglutamates and polyglutamylated 5-methyltetrahydrofolate (5-CH_3_-H_4_PteGlu_2−4_) in *Bifidobacterium adolescentis* DSM 20083^T^ and *Bifidobacterium pseudocatenulatum* DSM 20438^T^ *in vitro*. Folates were measured by means of stable isotope dilution assays (SIDA) coupled with LC-MS/MS analysis using [^2^H_4_]-5-methyltetrahydrofolic acid, [^2^H_4_]-tetrahydrofolic acid, and [^2^H_4_]-5-formyltetrahydrofolic acid as internal standards. Cell viability was examined by fluorescence microscopy. Quantitation of folate production by *B. adolescentis* during the stationary phase revealed a linear increase of dead cells paralleled by increasing concentration of 5-formyltetrahydrofolate and 5-methyltetrahydrofolate (100% 5-CH_3_-H_4_PteGlu_4_) in FFM, whereas the intracellular concentrations of these vitamers remained constant. After 24 h, *B. adolescentis* (125 mg cells, wet weight) produced a total amount of 0.846 nmol 5-CH_3_-H_4_folate: 0.385 ± 0.059 nmol (46 ± 7%) and 0.461 ± 0.095 nmol (54 ± 11%) measured in the intracellular (viable cells; 52 ± 3% measured by fluorescence microscopy) and extracellular (lysed cells; 48 ± 3%) fraction, respectively. For *B. pseudocatenulatum* (124 mg cells, wet weight), 1.135 nmol 5-CH_3_-H_4_folate was produced after 24 h, and a similar proportionality between intra- and extra-cellular folate concentrations and viable/lysed cells was observed. These results indicate that the strains tested produce and accumulate 5-CH_3_-H_4_PteGlu_4_ for cellular metabolism, and that extracellular concentrations of the vitamer arise from cell lysis.

## Introduction

To the best of our knowledge, only a few attempts have been made to characterize folate production patterns by bifidobacteria *in vitro*: Pompei et al. (2007) analyzed the intra- and extra-cellular total folate content after 48 h cultivation in folate-free media (FFM) by quantifying the growth of folate-dependent bacteria in a so-called microbiological assay (MA). D'Aimmo et al. (2012) investigated intracellular folate production and folate patterns in certain bifidobacteria strains by high performance liquid chromatography coupled with UV and fluorescence detection (HPLC-UV/FD). The latter authors identified 5-CH_3_-H_4_folate (structures of folate vitamers, see Figure S1) and H_4_folate as main vitamers accumulated by cells cultivated in FFM.

We recently developed a stable isotope dilution assay (SIDA) coupled with LC-MS/MS for sensitive quantitation of the main folate vitamers accumulated and released by *Bifidobacterium adolescentis* DSM 20083^T^, including 5-CH_3_-H_4_folate, 5-HCO-H_4_folate, H_4_folate, and 5-CH_3_-H_4_PteGlu_2−4_ (Kopp et al., 2016). Moreover, we found that the major portion of native 5-CH_3_-H_4_folate vitamer was present as its tetraglutamate (>95%) intra- and extra-cellularly. Concentrations of mono-, di-, tri-, and penta-glutamylated folates were below limit of detection (LOD) or limit of quantitation (LOQ).

From these results, the question arose about the possible origin of extracellular folates and particularly polyglutamylated forms. According to the literature (Deguchi et al., 1985; Pompei et al., 2007), intracellularly synthesized folate vitamers can be excreted into the cultivation medium. However, this hypothesis has to date not been tested experimentally and appears very unlikely as folate vitamers with three or more glutamate residues are fully retained by cells based on observations in *Lactobacillus casei* (Shane and Stokstad, 1975). Extracellular folates may indeed originate from intracellular folates, but without being actively excreted and rather via liberation from bacterial cells after their death and desintegration.

To test this hypothesis, microbiological, and analytical methods were applied to quantify folates and cell viability using pure cultures of *B. adolescentis* DSM 20083^T^ and *Bifidobacterium pseudocatenulatum* DSM 20438^T^. We examined intra- and extra-cellular native polyglutamate patterns as well as pteroylmonoglutamate concentrations after deconjugation of polyglutamates, and calculated the absolute amount [nmol] of monoglutamate in cell biomass and defined volumes of FFM. If the assumptions of passive release were correct, two criteria have to be fulfilled: (criterion 1) the intra- and extra-cellular degree of vitamer-specific polyglutamylation with three (retention in cells occurs with a minimum of three glutamate residues) or more glutamate residues should be identical and (criterion 2) the ratio of the absolute intra- and extra-cellular amounts compared to the total amount produced should reflect the relative viability of the strains. In this regard, dead (i.e., not cultivable) but intact cells might still be able to produce folates and, thus have to be considered as viable cells. For confirmation the relative viability was determined by fluorescence microscopy as independent microbiological method.

## Materials and methods

### Chemicals

All chemicals used for cultivation of bifidobacteria, reagents for extraction, and LC-MS/MS solvents have been published earlier (Kopp et al., 2016). Rat serum (preservative free) was obtained from Biozol (Eching, Germany). Chicken pancreas was purchased from Becton Dickinson and Co. (Sparks, MD, USA). 5-CH_3_-H_4_folate, 5-formyltetrahydrofolic acid (5-HCO-H_4_folate) calcium salt, and H_4_folate trihydrochloride were purchased from Schircks Laboratories (Jona, Switzerland). The isotopologic standards [^2^H_4_]-5-CH_3_-H_4_folate, [^2^H_4_]-5-HCO-H_4_folate, and [^2^H_4_]-H_4_folate were synthesized as reported recently (Freisleben et al., 2002). 5-CH_3_-H_4_PteGlu_2−4_ were synthesized according to Ndaw et al. (2001).

### Materials

Strata strong anion exchange (SAX) cartridges (100 mg, 1 mL) were obtained from Phenomenex (Aschaffenburg, Germany). AnaeroGen 2.5 L (GasPak) was obtained from Oxoid (Hampshire, UK). Chemicals for FFM were mixed according to D'Aimmo et al. (2012). LIVE/DEAD BacLight Bacterial Viability Kit L7012 for fluorescence microscopy was obtained from Molecular Probes (Eugene, Oregon, USA). For fluorescence microscopy we used an Axiostar plus (HBO 50) microscope with a red BP 546/12; FT 580; LP 590 and a green BP 475/40; FT 500; BP 530/50 fluorescence filter from Carl Zeiss Microimaging GmbH (Göttingen, Germany). Zirconium beads (0.1 mm) were obtained from Carl Roth GmbH (Karlsruhe, Germany). The bead beater Fast Prep 24 was manufactured by MP Biomedicals (Solon, OH, USA). Petri plates were purchased from Sarstedt (Nümbrecht, Germany). The HLC Thermomixer was manufactured by DITABIS AG (Pforzheim, Germany).

### Cultivation of bifidobacteria

*B. adolescentis* DSM 20083^T^ and *B. pseudocatenulatum* DSM 20438^T^ were obtained in dried form from the German Collection of Microorganisms and Cell Cultures (DSMZ, Braunschweig, Germany).

All glass devices and materials were autoclaved prior to use. Experiments were carried out under a laminar flow cabinet. Incubation of the anaerobic bacteria in Falcon tubes was carried out in an anaerobic jar with activated GasPak (Oxoid). MRS medium consisted of 26 g MRS bouillon in 487.5 mL water and was autoclaved at 121°C for 20 min. A volume of 12.5 mL 2% (w/v) L-cysteine solution was added as reducing agent. For long-term storage, bacteria were stored at −80°C in filter-sterilized glycerol (20% w/v) in MRS. Chemicals for FFM medium were mixed according to D'Aimmo et al. (2012).

For each experiment, one cryo-aliquot (200 μl) was inoculated into 25 mL medium and incubated for 24 h at 37°C. Afterwards, 1.25 mL was used for inoculating fresh medium to a final dilution ratio of 5% (preculture). After 24 h at 37°C, the working culture was prepared by diluting 0.5 mL preculture into 9.5 mL medium. The working culture was incubated for further 24 h at 37°C prior to extraction.

### Folate extraction

#### Solutions for folate extraction

Extraction buffer consisted of a 200 mmol·L^−1^ MES hydrate and 20 g·L^−1^ ascorbic acid aqueous solution with 1 g·L^−1^ DTT, adjusted to pH 5 with 7.5 M NaOH. Phosphate buffer (100 mmol·L^−1^) was prepared by adjusting an aqueous solution of disodium hydrogen phosphate (100 mmol·L^−1^) with an aqueous solution of potassium dihydrogen phosphate (100 mmol·L^−1^) to pH 7.0. The equilibration buffer for the SAX cartridges was prepared by adding 0.2 g·L^−1^ DTT to diluted phosphate buffer (10 mmol·L^−1^). Further, the eluting solution was a mixture of aqueous sodium chloride (5%) and aqueous sodium acetate (100 mmol·L^−1^) containing 1 g·L^−1^ DTT and ascorbic acid (1%). The chicken pancreas suspension for pteroylpolyglutamate deconjugation to the pteroyldiglutamates was prepared by stirring chicken pancreas (30 mg) in aqueous phosphate buffer solution (90 ml, 100 mmol·L^−1^) containing 1% ascorbic acid adjusted to pH 7 with 7.5 M NaOH. Rat serum was used for pteroyldiglutamate deconjugation to the monoglutamates without further dilution.

#### Extraction procedure

Bacteria suspension (culture volume 10 mL) was centrifuged at 5000 g for 10 min at 21°C. The supernatant was subjected to the extraction protocol below, whereas the cellular fraction was suspended in 2 mL of sterile water and divided into two aliquots. Aliquot 1 was subjected to fluorescence microscopy (Section Determination of Relative Viability by Fluorescence Microscopy), aliquot 2 was resuspended in 4 mL PBS, centrifuged for 15 min at 5000 g and 4°C. The supernatant was discarded and cellular fraction was resuspended in 10 mL extraction buffer and subjected to monoglutamate or polyglutamate extraction as described recently (Kopp et al., 2016).

Endogenous folate was determined by performing the extraction procedure for intra- and extra-cellular pteroylmonoglutamates by replacing the sample by 1 mL of distilled water. Endogenous folate was subtracted from all relevant values after LC-MS/MS measurement.

### LC-MS/MS

Extra- and intra-cellular pteroylmono- and pteroylpoly-glutamates were determined separately by means of LC-MS/MS according to our method described previously (Kopp et al., 2016).

### Determination of relative viability by fluorescence microscopy

For determination of viable and dead cell counts, working cultures were incubated at 37°C for 24 h. The LIVE/DEAD BacLight Bacterial Viability Kit, which consists of SYTO 9 (3.34 mM in DMSO) and propidium iodide (20 mM in DMSO), was used. After incubation, bacterial suspensions were centrifuged at 5000 g for 10 min. Pellets were re-suspended in 2 mL of sterile distilled water. One milliliter of the suspension was diluted in 20 mL distilled water and left to stand for 60 min at room temperature. The suspension was shaken after 15, 30, and 45 min. The sample was subsequently centrifuged for 10 min at 5000 g at 21°C. After resuspension in 20 mL distilled water and further centrifugation, the pellet was diluted in 10 mL of sterile water. One milliliter was mixed with 3 μL of the color mixture consisting of SYTO 9 and propidium iodide (50/50 v/v). The cells were incubated at room temperature in the dark for 15 min. Three aliquots were counted four times each under a microscope with a green (BP 475/40; FT 500; BP 530/50) and red (BP 546/12; FT 580; LP 590) fluorescence filter.

Relative viabilities [%] were calculated by dividing green (viable) and red (dead) cells by the total cell count multiplied by 100. Initial viabilities at t_0_ = 0 min (removal from medium) were calculated from the values obtained from fluorescence microscopy (t_1_) using the strain-specific calibration functions for mortality during sample preparation (see Supplementary Section S1, Table S1, and Figure S2). Therefore, the duration of the washing step in distilled water was considered, i.e., the time (t_1_−t_0_) between removing the culture from FFM and fluorescence microscopy.

### Extra- and intra-cellular folate production of *B. adolescentis* DSM 20083^T^ during stationary phase

Growth of *B. adolescentis* DSM 20083^T^ was determined by drop plate method to identify the stationary phase (see Supplementary Section S2). Six working cultures of *B. adolescentis* were incubated at 37°C. After 15, 19, 23, 37, 41, and 45 h, a single culture was removed and extracted in triplicate according to the procedure described above (Section Extraction Procedure). The respective amount of dead cells was determined by fluorescence microscopy (Section Determination of Relative Viability by Fluorescence Microscopy).

### Characterization of folate production by *B. adolescentis* DSM 20083^T^ and *B. pseudocatenulatum* DSM 20438^T^

To clarify whether 5-CH_3_-H_4_folate is excreted into the medium or originates from cell lysis, we determined the degree of 5-CH_3_-H_4_folate polyglutamylation, intra- and extra-cellular 5-CH_3_-H_4_folate and the percental amount of viable and dead cells by fluorescence microscopy. Growth of *B. pseudocatenulatum* DSM 20438^T^ was recorded using the drop plate technique to determine a defined time point for accurate sampling (see Supplementary Section S2).

One working culture of each strain was incubated for 24 h at 37°C. Supernatant and cellular fraction were separated as described above (Section Extraction Procedure). After resuspension in 2 mL of distilled water, 1 mL of the cellular fraction was subjected to fluorescence microscopy. The remaining aliquot and the supernatant were analyzed in triplicate for monoglutamate and polyglutamate patterns according to the extraction procedure (Section Extraction Procedure). After LC-MS/MS analysis 5-CH_3_-H_4_folate and 5-CH_3_-H_4_PteGlu_2−4_ patterns were compared to calculate the percental amount of each polyglutamate. Relative viability and relative mortality were calculated on the basis of the cellular fraction suspended in 2 mL distilled water (Section Determination of Relative Viability by Fluorescence Microscopy) and the supernatant (total volume of 10 mL) using 5-CH_3_-H_4_folate (A) as marker compound:

$$\begin{array}{l} n\left(A,\;intracellular,LC/MS\right)\left[nmol\right]\\ +n\left(A,\;extracellular,LC/MS\right)\left[nmol\right]\\ =n\left(A,\;total,LC/MS\right)\left[nmol\right]\\ \frac{n\left(A,\;intracellular,LC/MS\right)\left[nmol\right]}{n\left(A,\;total,LC/MS\right)\left[nmol\right]}*100\%\\ =relative\;viability\left(calculated\right)\;\left[\%\right]\;\left(after\;24\;h\right)\\ 100\%-relative\;viability\left(calculated\right)\;\left[\%\right]\\ =relative\;mortality\left(calculated\right)\;\left[\%\right]\left(after\;24\;h\right) \end{array}$$

In analogy we calculated the intra- and extra-cellular amount of 5-CH_3_-H_4_folate from the total amount of 5-CH_3_-H_4_folate by multiplication with the corrected, percental viable and dead cell count (see Supplementary Section S1) obtained from fluorescence microscopy (FM).

$$\begin{array}{l} n\left(A,total,LC/MS\right){\left[nmol\right]}^{*}relative\;viability\left(FM\right)\;\left[\%\right]\\ =n\left(A,\;intracellular,calculated\right)\left[nmol\right]\\ n\left(A,total,LC/MS\right)\left[nmol\right]\\ -n\left(A,\;intracellular,calculated\right)\left[nmol\right]\\ =n\left(A,extracellular,calculated\right)\left[nmol\right] \end{array}$$

### Data analysis

Data analysis was carried out using the Xcalibur Software version 2.0 (Thermo Scientific, Waltham, USA). Student's *t*-test was used for statistical comparisons, with *p* < 0.05 as threshold for significance.

## Results

Bacteria like *L. casei* have been well-characterized for intra-cellular folate accumulation, retaining pteroylpolyglutamates with three or more glutamate residues (Shane and Stokstad, 1975). In contrast, mono- or di-glutamylated folates may pass the cell membrane. To test the hypothesis that extracellular folate is solely a remnant of cell lysis, two experiments were performed. We examined extracellular enrichment and intracellular accumulation of 5-HCO-H_4_folate, H_4_folate, 5-CH_3_-H_4_folate and its polyglutamylated analogs (5-CH_3_-H_4_PteGlu_2−4_) in *B. adolescentis* DSM 20083^T^ during the stationary phase. Furthermore, we investigated the folate production and release by *B. adolescentis* DSM 20083^T^ and *B. pseudocatenulatum* DSM 20438^T^ after 24 h cultivation using 5-CH_3_-H_4_folate and 5-CH_3_-H_4_PteGlu_2−4_ as marker compounds for cellular transport.

### Extra- and intra-cellular folate production of *B. adolescentis* DSM 20083^T^ during the stationary phase

**Figure 1:**
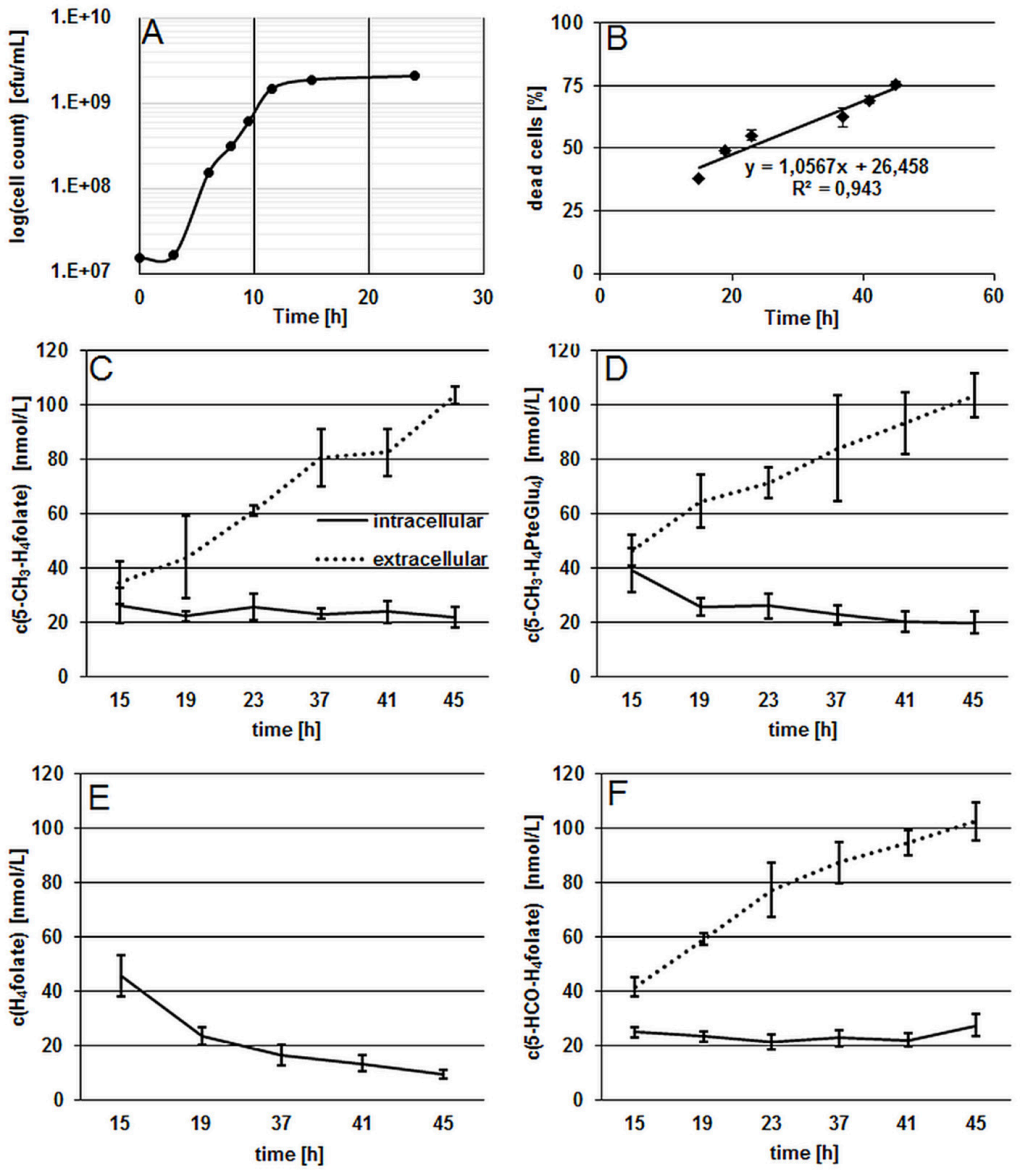
**Characterization of growth and folate production in *B. adolescentis* DSM 20083^T^ during the stationary phase. (A)** Growth curve of *B. adolescentis* DSM 20083^T^ in FFM determined by the drop plate method (cells counted two times), measurement uncertainty 6.1%. **(B)** Linear increase of dead cells during the stationary phase. **(C,D)** Intra- and extra-cellular folate concentrations of 5-CH_3_-H_4_folate **(C)** and 5-CH_3_-H_4_PteGlu_4_ **(D)** depicted as means of triplicate extraction. **(E)** Intra-cellular concentrations of H_4_folate. **(F)** Intra- and extra-cellular concentrations of 5-HCO-H_4_folate.

Results from the growth experiment of *B. adolescentis* DSM 20083^T^ in FFM showed a fast adaption to the synthetic medium which is depicted by the short lag-phase from 0 to 3 h (Figure 1A).

The stationary phase was reached after 11.5 h and viable cell counts remained constant after 15 h. In the following experiment, the ratio of dead cells showed a linear increase from 15 to 45 h during the stationary phase (Figure 1B), confirming that steady state conditions were applied for folate analysis. As the stationary phase is characterized by a constant number of viable cells and an increasing number of dead cells, folate content should increase in the extracellular fraction and remain approximately constant in cells according to our hypothesis. As depicted in Figures 1C–F, we measured the three main monoglutamylated folate vitamers and 5-CH_3_-H_4_PteGlu_4_, identified as the predominant polyglutamylated 5-CH_3_-H_4_folate in both cells and FFM (Kopp et al., 2016), between 15 and 45 h of incubation. The further vitamer 5-HCO-H_4_folate (Figure 1F) showed curve shape and concentrations similar to that of 5-CH_3_-H_4_folate. For the least stable folate vitamer, H_4_folate, we observed a significant decrease (*P* < 0.05) intracellularly during steady state conditions (Figure 1E).

### Characterization of folate production by *B. adolescentis* DSM 20083^T^

One culture of *B. adolescentis* DSM 20083^T^ was examined for intra- and extra-cellular 5-CH_3_-H_4_folate and 5-CH_3_-H_4_PteGlu_2−4_ after 24 h incubation in FFM. 5-CH_3_-H_4_folate and 5-CH_3_-H_4_PteGlu_2−4_ were used as marker compounds for folate accumulation and release because of their chemical stability compared to H_4_folate and 5-HCO-H_4_folate, which tend to be oxidized or interconverted. Our primary goal was to clarify whether folate is transferred into the medium by either excretion or cell lysis. Therefore, we compared the amount of extra- and intracellular 5-CH_3_-H_4_folate obtained from direct measurement by SIDA LC-MS/MS with the amount of folate as extrapolated from relative cell viability measured (see Section Materials and Methods).

**Figure 2:**
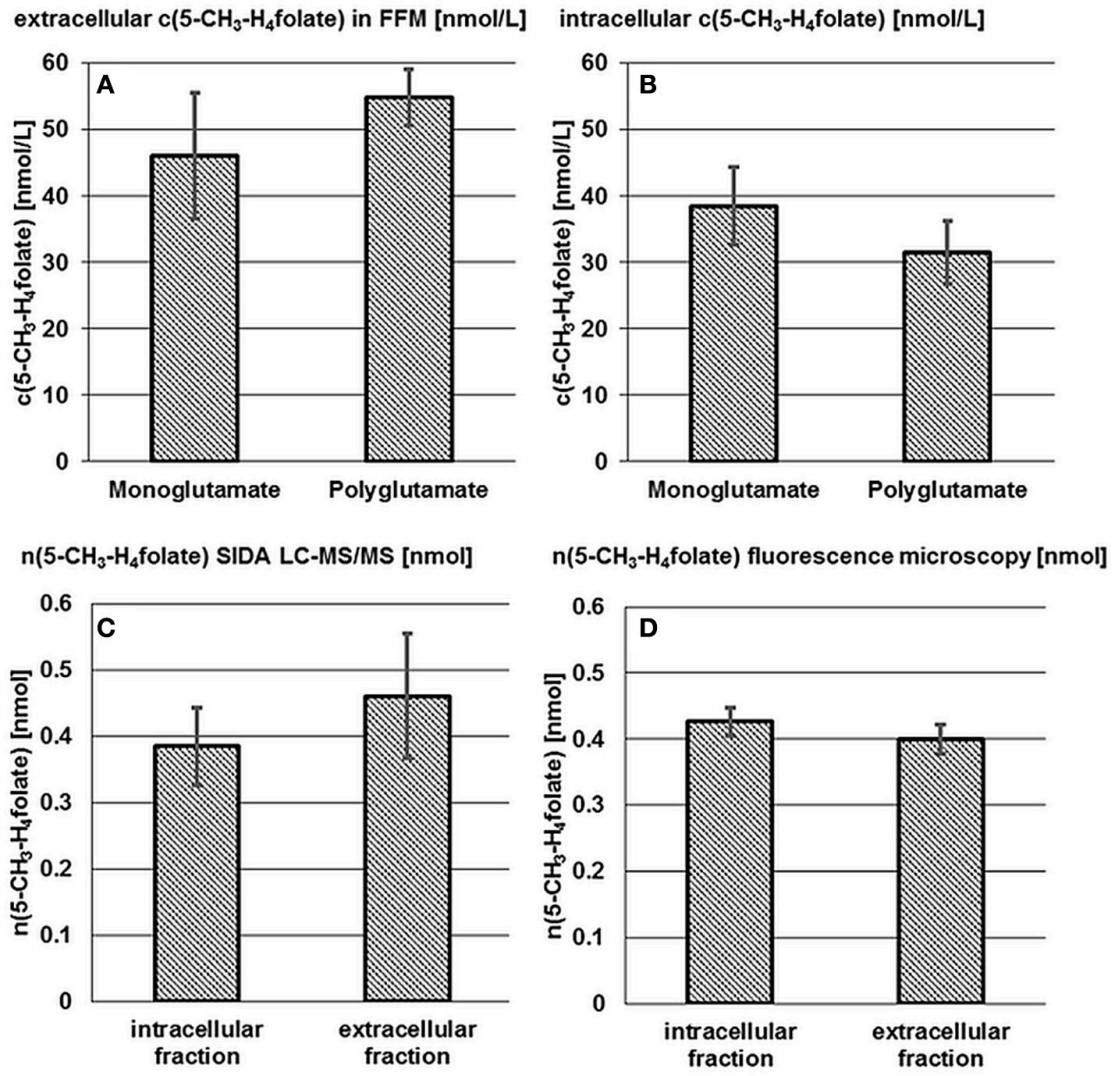
**Accumulation and release of 5-CH_3_-H_4_folate by *B. adolescentis* DSM 20083^T^**. Extra- **(A)** and intracellular **(B)** 5-CH_3_-H_4_folate concentrations in cultures of *B. adolescentis* DSM 20083^T^ after 24 h in FFM. Absolute amount of 5-CH_3_-H_4_folate calculated from SIDA LC-MS/MS **(C)** and by extrapolation from fluorescence microscopy **(D)**. Data are shown as mean and standard deviations (triplicate experiments).

The term “monoglutamate” is used below in connection with the total amount of the respective vitamer after enzymatic deconjugation. The term “polyglutamate” refers to the native vitamer and its degree of polyglutamylation without deconjugation. Results of mono- and poly-glutamate analysis are shown in Figures 2A,B.

As there is hardly any difference between the mono- and poly-glutamate contents, almost 100% of 5-CH_3_-H_4_folate can be ascribed to 5-CH_3_-H_4_PteGlu_4_ after 24 h which is consistent with the results from the steady state experiment (Section Extra- and Intra-cellular Folate Production of *B. adolescentis* DSM 20083^T^ during the Stationary Phase).

An intracellular amount of n(5-CH_3_-H_4_folate,LC/MS)_intracellular_ = 0.385 ± 0.059 nmol was determined by LC-MS/MS (Figure 2C). For the extracellular fraction n(5-CH_3_-H_4_folate,LC/MS)_extracellular_ = 0.461 ± 0.095 nmol were determined (Figure 2D). Based on a total amount of n(5-CH_3_-H_4_folate,LC/MS)_total_ = 0.846 nmol a relative viability of 46 ± 7% and a relative mortality of 54 ± 11% were calculated from folate analysis (Table 1).

**Table 1:** **Relative viabilities obtained from fluorescence microscopy (measured) or SIVA-LC-MS/MS (calculated)**.

**Strain**	**Relative viability calculation (%)**	**Fluorescence microscopy**	**Ratio of absolute amounts 5-CH_3_-H_4_folate intra-/extra-cellular to total**
*B. adolescentis* DSM 20083^T^	Viable cells	52 ± 3	46 ± 7
	Dead cells	48 ± 3	54 ± 11
*B. pseudocatenulatum* DSM 20438^T^	Viable cells	56 ± 3	56 ± 7
	Dead cells	44 ± 3	44 ± 10

*B. adolescentis* showed a strong correlation between time and viability in distilled water during cell purification. Therefore, the duration of the washing step (t_1_−t_0_, min) between removal of the centrifuged bacteria from the culture (t_0_) and fluorescence microscopy (t_1_) was multiplied with the slope of the calibration function (−0.0865; Figure S2A). From fluorescence microscopy (Figure S3), which showed good precision with CVs of 3%, we obtained 52 ± 3% viable and 48 ± 3% dead cells after correcting for the washing step (Table 1).

These results were multiplied with the sum of 0.846 nmol total folate in the whole culture assay (Figure 2D, Table S2). Finally, comparable amounts were obtained with n(5-CH_3_-H_4_folate,calculated)_intracellular_ = 0.426 ± 0.022 nmol and n(5-CH_3_-H_4_folate,calculated)_extracellular_ = 0.400 ± 0.022 nmol. Both methods showed no significant difference of the absolute amount of intra- and extra-cellular 5-CH_3_-H_4_folate.

These results disprove the assumption that 5-CH_3_-H_4_folate is excreted actively by *B. adolescentis*, as it would imply a significant higher proportion of extracellular 5-CH_3_-H_4_folate to total 5-CH_3_-H_4_folate than the ratio of dead cells measured by microscopy. To substantiate these findings, we analyzed folate production by one additional species, *B. pseudocatenulatum*.

### Characterization of folate production by *B. pseudocatenulatum* DSM 20438^T^

**Figure 3:**
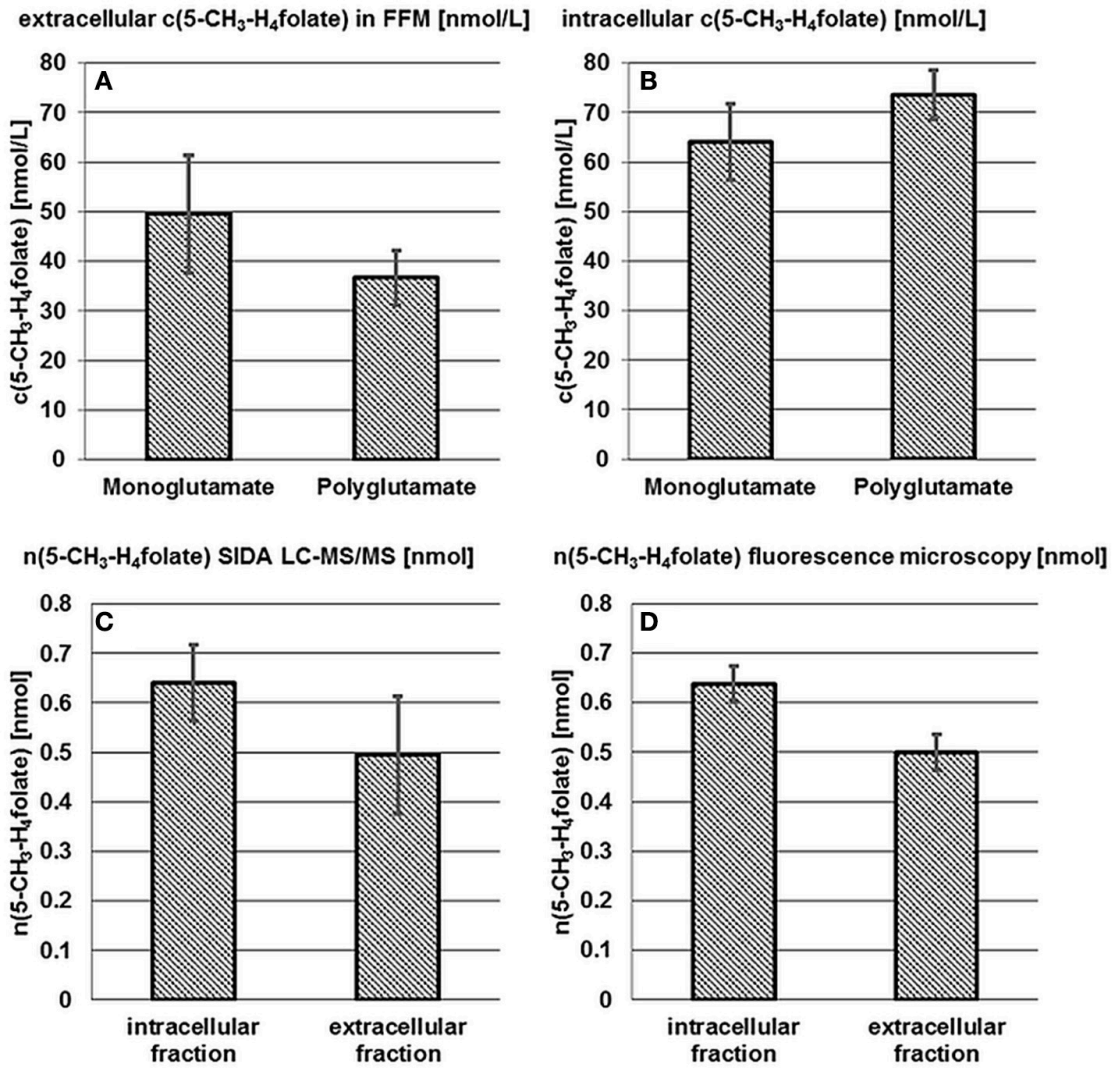
**Accumulation and release of 5-CH_3_-H_4_folate by *B. pseudocatenulatum* DSM 20438^T^**. See Figure 2.

*B. pseudocatenulatum* DSM 20438^T^ was examined for intra- and extra-cellular 5-CH_3_-H_4_folate and 5-CH_3_-H_4_PteGlu_2−4_ after 24 h incubation in FFM. Results of the mono- and poly-glutamate analysis are shown in Figures 3A,B.

50 ± 12 nmol·L^−1^ 5-CH_3_-H_4_folate and 37 ± 5.5 nmol·L^−1^ of the polyglutamylated vitamers were determined in the extracellular fraction (A) whereas the cells contained 64 ± 7.7 nmol·L^−1^ monoglutamate and 74 ± 5.0 nmol·L^−1^ polyglutamate (B). Thus and analogously to *B. adolescentis* almost 100% of 5-CH_3_-H_4_folate can be traced back to the tetraglutamate. No significant difference (*P* > 0.05) was found for the mono- and poly-glutamate content.

Based on LC-MS/MS measurements n(5-CH_3_-H_4_folate,LC/MS)_intracellular_ = 0.640 ± 0.077 nmol, n(5-CH_3_-H_4_folate,LC/MS)_extracellular_ = 0.495 ± 0.119 nmol (Figure 3C) and a total amount of n(5-CH_3_-H_4_folate,LC/MS)_total_ = 1.135 nmol were determined for cells, FFM and the sample as a whole, respectively. In analogy to *B. adolescentis* DSM 20083^T^ a relative viability of 56 ± 7% and a relative mortality of 44 ± 10% was calculated using the LC-MS approach.

A relative, corrected (Figure S2B) amount of 56 ± 3% viable and 44 ± 3% dead cells was determined by fluorescence microscopy. *B. pseudocatenulatum* formed small cell clusters and cell distribution was less homogenous than observed for *B. adolescentis*. Nevertheless, CVs for viability and the ratio of dead cells were 3%. After multiplication of the relative viability with the total amount of 1.135 nmol 5-CH_3_-H_4_folate (Figure 3D, Table S3) n(5-CH_3_-H_4_folate,calculated)_intracellular_ = 0.637 ± 0.036 nmol and n(5-CH_3_-H_4_folate,calculated)_extracellular_ = 0.498 ± 0.036 nmol were calculated for cellular fraction and FFM, respectively. Both methods showed no significant (*P* > 0.05) difference between the absolute amount of intra- and extra-cellular 5-CH_3_-H_4_folate.

Compared to the results obtained with *B. adolescentis*, we found an even higher agreement between viable/dead cells and intra-/extra-cellular folate for *B. pseudocatenulatum*.

## Discussion

Characterization of folate synthesis by bacteria is challenging as analytical methodologies for folate determination differ considerably and imprecision of measurements might lead to a misinterpretation of results.

Our previously developed SIDA LC-MS/MS for detailed assessment of intra- and extra-cellular folate patterns in bifidobacteria (Kopp et al., 2016) showed a high reproducibility for main folate vitamers as well as an accurate quantitation of mono- and polyglutamylated 5-CH_3_-H_4_folate. In the present manuscript, fluorescence microscopy was used for determination of viable/dead cell ratios.

Stationary phase experiments with *B. adolescentis* DSM 20083^T^ revealed equivalent intra- and extra-cellular folate patterns of 5-CH_3_-H_4_folate, depicted by the fact that 5-CH_3_-H_4_folate can be traced back solely to its tetraglutamate. Moreover, 5-HCO-H_4_folate and 5-CH_3_-H_4_folate showed similar intra- and extra-cellular curve progressions with an increasing vitamer concentration in FFM and a constant concentration in the cellular fraction. Nevertheless, 5-HCO-H_4_folate has to be regarded as sum of non-methylated folates like 5,10-CH^+^-H_4_folate, 5-HCO-H_4_folate, and 10-HCO-H_4_folate being prone to interconversion (May et al., 1951; Wilson and Horne, 1983; Brouwer et al., 2007), whereas 5-CH_3_-H_4_folate remains stable. In contrast to 5-HCO-H_4_folate and 5-CH_3_-H_4_folate, H_4_folate was not detected extracellularly, which might be attributed to its low stability. Intracellular H_4_folate decreased significantly under steady state conditions. In growing cells, H_2_folate and H_4_folate are the first folate vitamers synthesized (Rossi et al., 2011). After folate polyglutamylation functional groups are transferred for essential metabolic reactions like DNA synthesis. The decrease in H_4_folate might be attributed to the fact that initially high synthesis rates of H_4_folate related to immense growth during the exponential phase are reduced because of a reduced availability of nutritional factors in the medium. H_4_folate might be converted to essential biological active folate vitamers as they are necessary to maintain growth during the stationary phase.

Nevertheless, we were able to prove the similarity of intra- and extra-cellular folate patterns (criterion 1) by showing that a linear increase in dead cells, followed by an enrichment of 5-CH_3_-H_4_folate in the extracellular fraction can be ascribed to ~100% 5-CH_3_-H_4_PteGlu_4_ whereas the concentration in cells remains constant. Therefore, the hypothesis of active folate excretion seems to be at least partly invalid. In the next experiment, we examined and compared the relative viability from fluorescence microscopy and the analytical approach to prove or disprove criterion (2). In our main experiments with *B. adolescentis* DSM 20083^T^ and *B. pseudocatenulatum* DSM 20438^T^, we found no extracellular H_4_folate, which is either not excreted or underlies increased degradation in FFM after cell lysis. Because of folate interconversion, 5-HCO-H_4_folate seems to be no specific marker as its content reflects the concentration of more than one vitamer. Therefore, we chose 5-CH_3_-H_4_folate as marker compound and determined intra- and extra-cellular concentrations of this vitamer and its polyglutamate patterns. The determination of 5-CH_3_-H_4_PteGlu_2−4_ revealed 5-CH_3_-H_4_PteGlu_4_ to be the only polyglutamic form released from cells into the medium (~100%) after 24 h. Combined with the relative count of viable and dead cells we obtained a significant correlation between viability and absolute amount of intra- and extra-cellular 5-CH_3_-H_4_folate. Because the ratio of the absolute intra- and extra-cellular amounts compared to the total amount produced by the strains is consistent with their relative viabilites after 24 h cultivation (criterion 2), we conclude for 5-CH_3_-H_4_folate that cell lysis is the only determinant for extracellular 5-CH_3_-H_4_folate enrichment *in vitro*. This conclusion is valid for the sensitivity range of our assay, which would detect mono- and poly-glutamates exceeding 10% of the total content of all 5-CH_3_-H_4_folate forms extracellularly.

Altogether, the results of the present work refute early findings assuming active excretion of folates by bifidobacteria (Deguchi et al., 1985; Pompei et al., 2007) and imply that 5-CH_3_-H_4_PteGlu_4_ is released via cell lysis, particularly in the stationary phase of the bacteria culture. This conclusion is further substantiated by the lack of evidence for any mechanisms of transport of polyglutamylated folate vitamers. Intestinal deconjugation of pteroylpolyglutamates is mediated by glutamate carboxypeptidase II (GPC II), a brush border transmembrane glycoprotein located in the proximal part of the jejunum (Reisenauer et al., 1977; Reisenauer and Halsted, 1981; McNulty and Pentieva, 2010). So far GPC II constitutes the only enzyme for pteroylpolyglutamate deconjugation identified in the human gut. Therefore, further experiments have to be performed to clarify the extent of colonic deconjugation which is crucial for colonic uptake of folate, as colonic folate production is assumed to contribute to the host nutritional status.

## Author contributions

MK, KD, MS, TC, and MR designed the studies. MK, KD, and MS developed and validated all methods. MK, KD, MS, and MR interpreted data. TC supervised microbiological experiments. MK, TC, and MR drafted the manuscript. All authors contributed to improvement of the manuscript and agreed with the content. MR had primary responsibility for funding and final manuscript content.

### Conflict of interest statement

The authors declare that the research was conducted in the absence of any commercial or financial relationships that could be construed as a potential conflict of interest.
