# Supplementary material for: Measurements of Intra- and Extra-Cellular 5-Methyltetrahydrofolate Indicate that Bifidobacterium Adolescentis DSM 20083T and Bifidobacterium Pseudocatenulatum DSM 20438T Do Not Actively Excrete 5-Methyltetrahydrofolate In vitro
Source: Front Microbiol. 2017 Mar 21;8:445. doi: 10.3389/fmicb.2017.00445 (PMC5359228; doi:10.3389/fmicb.2017.00445)
Supplement: Supplementary file 1 [file DataSheet1.DOCX]

***Supplementary material***

**Measurements of intra- and extracellular 5-methyltetrahydrofolate indicate that *Bifidobacterium adolescentis* DSM 20083^T^ and *Bifidobacterium pseudocatenulatum* DSM 20438^T^ do not actively excrete 5-methyltetrahydrofolate *in vitro***

Markus Kopp ^1,2^, Kerstin Dürr ^1^, Matthias Steigleder ^1^, Thomas Clavel ^2^, and Michael Rychlik ^1^*

*** Correspondence:** Michael Rychlik, [michael.rychlik@tum.de](mailto:michael.rychlik@wzw.tum.de)

**1. Supplementary data**

**S-1. Dependence of viable/dead cell ratio on purification time between removal from FFM and fluorescence microscopy**

For cell purification prior to fluorescence microscopy we used a standard procedure proposed by the manufacturer of the staining kit. The washing step includes the preparation of cell suspensions in distilled water. We assumed an increasing mortality rate during the repeated washing steps. Therefore, we quantified the degree of cellular degradation after defined washing periods and extrapolated the linear time-degradation function to the initial ratio of viable/dead cells.

Influence of cell purification on the viable/dead cell ratio was determined as a function of time. Three working cultures (I-III) of *B. adolescentis* DSM 20083^T^ and *B. pseudocatenulatum* DSM 20438^T^ were prepared. Relative viability of each working culture was determined at defined time points in triplicate. Viability of *B. adolescentis* DSM 20083^T^ and *B. pseudocatenulatum* DSM 20438^T^ was examined after 192 (I), 272 (II), 292 (II) and 397 (III) min and 150 (I), 160 (I), 265 (II), 275 (II), 360 (III) and 380 (III) min in distilled water, respectively. The initial viability was calculated by linear regression of the incubation experiment.

Results of fluorescence microscopy are expressed as mean of fourfold counting (Table S-1). The initial relative viable cell count is calculated depending on the time period between removal from FFM (t_0_=0 min) and fluorescence microscopy after (t_1_-t_0_) min (Table S-1). Linear regression and the decrease in relative viable cell count for *B. adolescentis* is shown in Fig. S-2A. At 0 min a relative amount of 57% viable and 43% dead cells was obtained for *B. adolescentis* in FFM.

In analogy we investigated the mortality of *B. pseudocatenulatum*. Results are shown in Table S-1 and Fig. S-2B. At 0 min a relative amount of 86% viable and 14% dead cells can be obtained for *B. pseudocatenulatum* in FFM.

Because of the inversed viable/dead cell ratio after cell purification the mathematical model was adopted for the main experiment to correct for the time needed for sample preparation.

**S-2. Growth characteristics of *B. adolescentis* DSM 20083^T^ and *B. pseudocatenulatum* DSM 20438^T^ in FFM**

13 g MRS bouillon was solved in 243.75 mL water. After the addition of 5 g agar the solution was autoclaved at 121°C for 20 min. 6.25 mL of sterile L-Cys solution was added as growth factor and the solution was chilled in a water bath to approximately 50°C. 20 mL of the solution were poured into sterile petri dishes. After gelation dishes were stored upside-down in a resealable plastic bag at room temperature.

For the growth curves bacteria working cultures were produced in duplicate for each time (0, 3, 6, 8, 9,5, 11,5, 15, and 24). The data points for the curves were determined in duplicate using the drop plate method. Determination of viable cell count (colony forming units per mL, cfu/mL) was carried out by plating various dilutions of the bacteria suspension with FFM from 10^-1^ to 10^-8^ on agar plates. 10 µL of each dilution were dropped in line and allowed to disperse after holding the plate in an inclined position. Incubation was carried out for 48 h at 37°C. After incubation colonies were counted and cfu/mL were calculated for each dilution.

**2. Supplementary figures and tables**

**Figure S-1:** Structures of folate vitamers produced by *B. adolescentis* DSM 20083^T^ and *B. pseudocatenulatum* DSM 20438^T^.

**Figure S-2:** Mortality rate of *B. adolescentis* DSM 20083^T^ **(A)** and *B. pseudocatenulatum* DSM 20438^T^ **(B)** depending on the duration of cell purification (t_1_-t_0_) between cellular removal from FFM (t_0_) and fluorescence microscopy (t_1_) (Table S-1). Data points are expressed as means of three experiments.

**
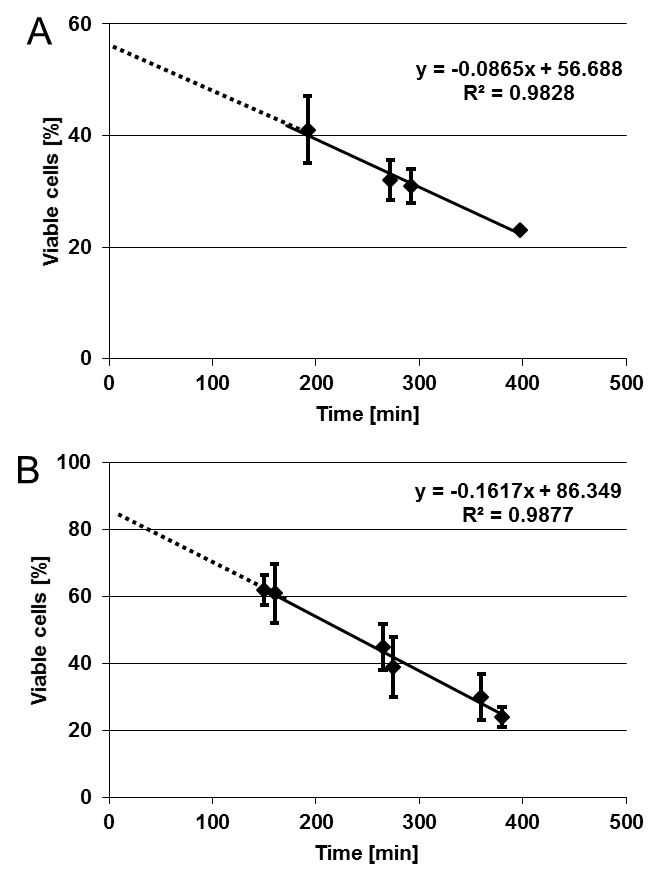
**

**Figure S-3: Fluorescence microscopy of *B. adolescentis* DSM 20083^T^ cultivated in FFM for 24 h.** Green (SYTO 9): Viable cells, red (propidium iodide): Dead cells. Magnification: 1:100.


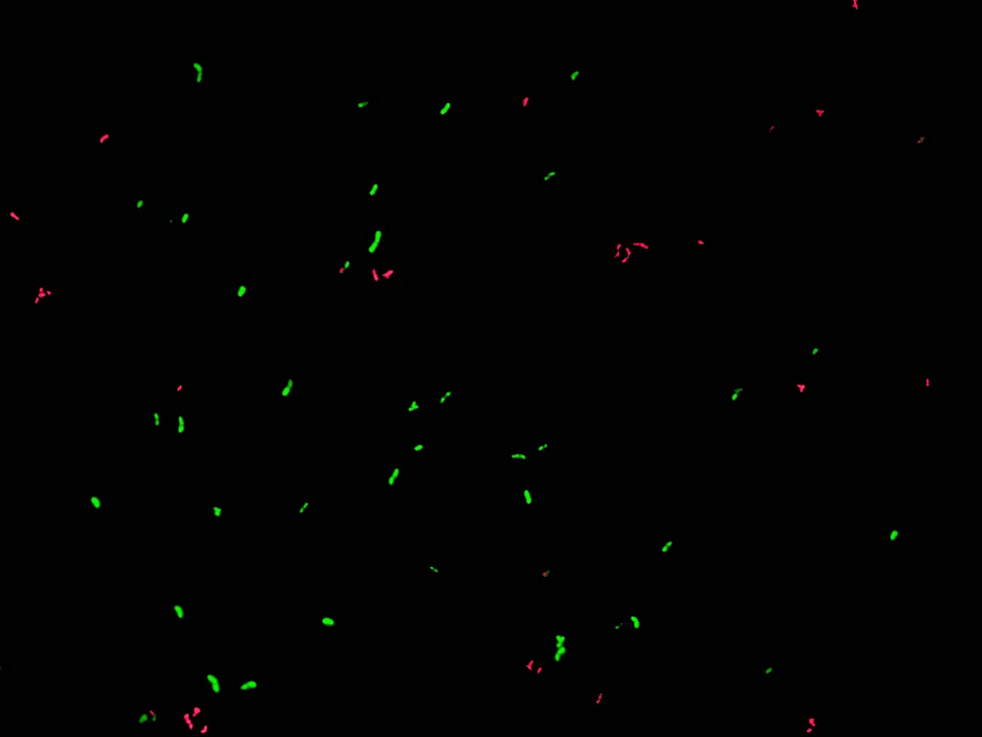


**Table S-1:** Influence of cell purification on cell mortality of *B. adolescentis* between cultivation and fluorescence microscopy.

| **strain** | **culture** | **time [min]** | **viable cells [%]** | **dead cells [%]** | **± SD [%]** |
| --- | --- | --- | --- | --- | --- |
| ***B. adolescentis***  **DSM 20083^T^** | I | 192 | 41 | 59 | 2 |
|  | II | 272 | 32 | 68 | 6 |
|  | II | 292 | 31 | 69 | 4 |
|  | III | 397 | 23 | 77 | 3 |
| ***B. pseudocatenulatum* DSM 20438^T^** | I | 150 | 62 | 38 | 5 |
|  | I | 160 | 61 | 39 | 9 |
|  | II | 265 | 45 | 55 | 7 |
|  | II | 275 | 39 | 61 | 9 |
|  | III | 360 | 30 | 70 | 7 |
|  | III | 380 | 24 | 76 | 3 |

**Table S-2: Viability of *B. adolescentis* DSM 20083^T^ determined by fluorescence microscopy after correction for the washing step (t_1_-t_0_).** Absolute intra- and extracellular amounts calculated by multiplication of the corrected (corr.) percentages of viable and dead cells with the total amount of 0,846 nmol in the culture obtained from LC-MS/MS.

| Repetition | t_1_-t_0_ [min] | viable [%] | corr. viable [%]* | dead [%] | corr. dead [%]* | n(5-CH_3_-H_4_folat) intracellular [nmol] | n(5-CH_3_-H_4_folat) extracellular [nmol] |
| --- | --- | --- | --- | --- | --- | --- | --- |
| 1 | 135 | 37,8 | 48,8 | 62,2 | 51,2 | 0,403 | 0,423 |
| 2 | 155 | 42,6 | 55,6 | 57,4 | 44,4 | 0,459 | 0,367 |
|  | 155 | 40,6 | 53,6 | 59,4 | 46,4 | 0,443 | 0,383 |
|  | 155 | 40,5 | 53,5 | 59,5 | 46,5 | 0,442 | 0,384 |
| 3 | 165 | 35,4 | 49,4 | 64,6 | 50,6 | 0,408 | 0,418 |
|  | 165 | 35,7 | 49,7 | 64,3 | 50,3 | 0,411 | 0,415 |
|  | 165 | 36,6 | 50,6 | 63,4 | 49,4 | 0,418 | 0,408 |
| mean±SD |  |  | **52±3** |  | **48±3** | **0,426±0,022** | **0,400±0,022** |

* corrected viability (Fig. S-1A)

**Table S-3: Viability of *B. pseudocatenulatum* DSM 20438^T^ determined by fluorescence microscopy after correction for the washing step (t_1_-t_0_).** Absolute intra- and extracellular amounts of 5-CH_3_-H_4_folate calculated by multiplication of the corrected (corr.) percentages of viable and dead cells with the total amount of 1,135 nmol in the culture obtained from LC-MS/MS.

| Repetition | t_1_-t_0_ [min] | viable [%] | corr. viable [%]* | dead [%] | corr. dead [%]* | n(5-CH_3_-H_4_folat) intracellular [nmol] | n(5-CH_3_-H_4_folat) extracellular [nmol] |
| --- | --- | --- | --- | --- | --- | --- | --- |
| 1 | 135 | 28,6 | 50,6 | 71,4 | 49,4 | 0,574 | 0,561 |
|  | 135 | 30,8 | 52,8 | 69,2 | 47,2 | 0,599 | 0,536 |
|  | 135 | 33,3 | 55,3 | 66,7 | 44,7 | 0,628 | 0,507 |
| 2 | 155 | 36,8 | 61,8 | 63,2 | 38,2 | 0,702 | 0,433 |
|  | 155 | 31,3 | 56,3 | 68,8 | 43,8 | 0,639 | 0,497 |
|  | 155 | 30,8 | 55,8 | 69,2 | 44,2 | 0,633 | 0,502 |
|  | 155 | 30,0 | 55,0 | 70,0 | 45,0 | 0,625 | 0,511 |
|  | 155 | 30,8 | 55,8 | 69,2 | 44,2 | 0,633 | 0,502 |
| 3 | 165 | 34,5 | 61,5 | 65,5 | 38,5 | 0,698 | 0,437 |
|  | 165 | 30,8 | 57,8 | 69,2 | 42,2 | 0,656 | 0,480 |
|  | 165 | 26,9 | 53,9 | 73,1 | 46,1 | 0,612 | 0,523 |
|  | 165 | 29,4 | 56,4 | 70,6 | 43,6 | 0,641 | 0,495 |
| mean±SD |  |  | **56±3** |  | **44±3** | **0,637±0,036** | **0,498±0,036** |

* corrected viability (Fig. S-1B)
